# Continuous targeted kinase inhibitors treatment induces upregulation of PD-L1 in resistant NSCLC

**DOI:** 10.1038/s41598-018-38068-3

**Published:** 2019-03-06

**Authors:** Li Jiang, Fuchun Guo, Xiaoke Liu, Xiaoyu Li, Qing Qin, Pei Shu, Yi Li, Yongsheng Wang

**Affiliations:** 1 Departments of Thoracic Oncology, Cancer Center, West China Hospital, Chengdu, Sichuan Province, 610041 China; 2 State Key Laboratory of Biotherapy, Sichuan University, Chengdu, Sichuan Province, 610041 China

## Abstract

First-generation epidermal growth factor receptor (EGFR) targeted kinase inhibitors (TKIs) are still used in selected non-small cell lung cancer (NSCLC) patients despite the resistance. Based on the correlation of programmed cell death receptor ligand 1 (PD-L1) and EGFR signaling pathway, whether continuous TKIs treatment will affect PD-L1 expression after disease progression remains unclear. To investigate the potential change of PD-L1 expression in TKI-resistant NSCLC after continuous TKIs treatment, we treated H1975 and HCC827 for more than one month and explored the possible effect on immune cells as well as underlying biological mechanisms. We found that continuous exposure to TKIs induced upregulation of PD-L1 in H1975 and HCC827. Moreover, PD-L1 upregulation significantly inhibited proliferation and slightly promoted apoptosis of T cells. We observed the activation of STAT3 and ERK1/2 along with the PD-L1 upregulation. With the pathway inhibitors, we found ERK1/2 pathway involved in inducing PD-L1 in resistant lung cancer. This study provides preclinical evidence that continuous TKIs treatment may induce PD-L1 expression in resistant NSCLC, resulting in the suppression of T cell function and immune escape. ERK1/2 pathway inhibitors, PD-L1/PD-1 inhibitors or combination strategies should be considered to reverse the resistance to TKIs in NSCLC patients.

## Introduction

Lung cancer remains the leading health challenge to humanity worldwide, with the second highest incidence and the highest mortality in both males and females^1^. It is still urgent to optimize therapy strategies for patients with advanced disease. Currently, 83% of lung cancers are classified as non-small cell lung cancer (NSCLC), most of which are at an advanced stage when the first diagnosis is performed. Chemotherapy with or without radiation therapy used to be the standard resolution, but in recent decade targeted kinase inhibitors (TKIs) are proved to be superior, especially in the oncogene-driven tumors, such as epidermal growth factor receptor (EGFR) or anaplastic lymphoma kinase (ALK)^2–5^. EGFR, also named HER1/erbB1, is a critical member of the HER/erbB family of receptor tyrosine kinases (RTKs). About 85–90% mutations in the TK domain of EGFR are exon 19 deletions and exon 21 L858R mutations, resulting in constitutive phosphorylation of important tyrosine residues and activation of downstream signaling pathways (such as mitogen-activated protein kinase (MAPK), phosphoinositide 3-kinase (PI3K), signal transducer and activator of transcription(STAT))^6,7^. Tumors bearing these EGFR mutations are particular sensitive to EGFR TKIs compared to those with wild-type EGFR^8^. EGFR TKIs reversibly inhibit EGFR activity through competing with adenosine triphosphate (ATP) for binding to the receptor’s kinase pocket, thus blocking EGFR auto-phosphorylation. Unfortunately, widespread acquired resistance to TKIs always takes place within 6 to 12 months, which greatly restricts the long-term efficacy of these drugs. The most common mechanism of acquired resistance is a second EGFR mutation on threonine 790 in the ATP binding pocket, named T790M^9^. The T790M mutation increases the ATP affinity of the oncogenic L858R mutant and sterically interferences the binding of TKIs^10^.

Currently, new generation TKIs (such as Afatinib and Osimertinib), toxic therapy, immunotherapy or combination strategies are advocated to deal with this intricate situation^11,12^. However, whether initial TKI therapy should be continued in resistant NSCLC has been debated. The IMPRESS trial indicated the continuation of Gefitinib failed to prolong progression-free survival in resistant NSCLC when combined with platinum-based doublet chemotherapy^13^. On the contrary, a retrospective study showed survival benefit from EGFR-TKIs beyond progressive disease compared to cytotoxic chemotherapy^14^. The ASPIRATION trial suggested Erlotinib was feasible for selected patients after progression^15^. As a result, Gefitinib and Erlotinib are still utilized in some TKI-resistant NSCLC in spite of possible limited benefit.

As reported recently, the expression of programmed cell death receptor ligand 1 (PD-L1) could be induced by the oncogenic EGFR mutation and reduced apparently by EGFR TKIs in EGFR-driven tumor^16^. The PD-1/PD-L1 pathway transfers inhibitory immune signals, which can limit tumor-infiltrating CD4+ and CD8+ T cells and contribute to immune evasion^17^. Accordingly, Gefitinib and Erlotinib may have a notable influence on the PD-L1 expression through changing the downstream signal pathways of EGFR, such as MAPK, PI3K, Janus kinase (JAK)/STAT). However, despite the initial inhibition of PD-L1 in EGFR-driven tumor, very limited information is known about the effect of continuous TKIs treatment on PD-L1 expression when NSCLC become resistant to TKIs. Based on STAT3 activation after continuous TKI treatment in our previous research^18^, we hypothesized that PD-L1 expression will increase in resistant NSCLC with the continuation of TKIs. To test the hypothesis, we treated H1975 and HCC827 for more than one month and tended to explore the possible effect on immune cells and the underlying biological mechanisms. Identifying the possible change of immune checkpoint will provide important information before clinical treatment strategies are made.

## Results

### Continuous exposure to TKIs induces upregulation of PD-L1 in H1975 (EGFR L858R/T790M) ***in vitro***

**Figure 1:**
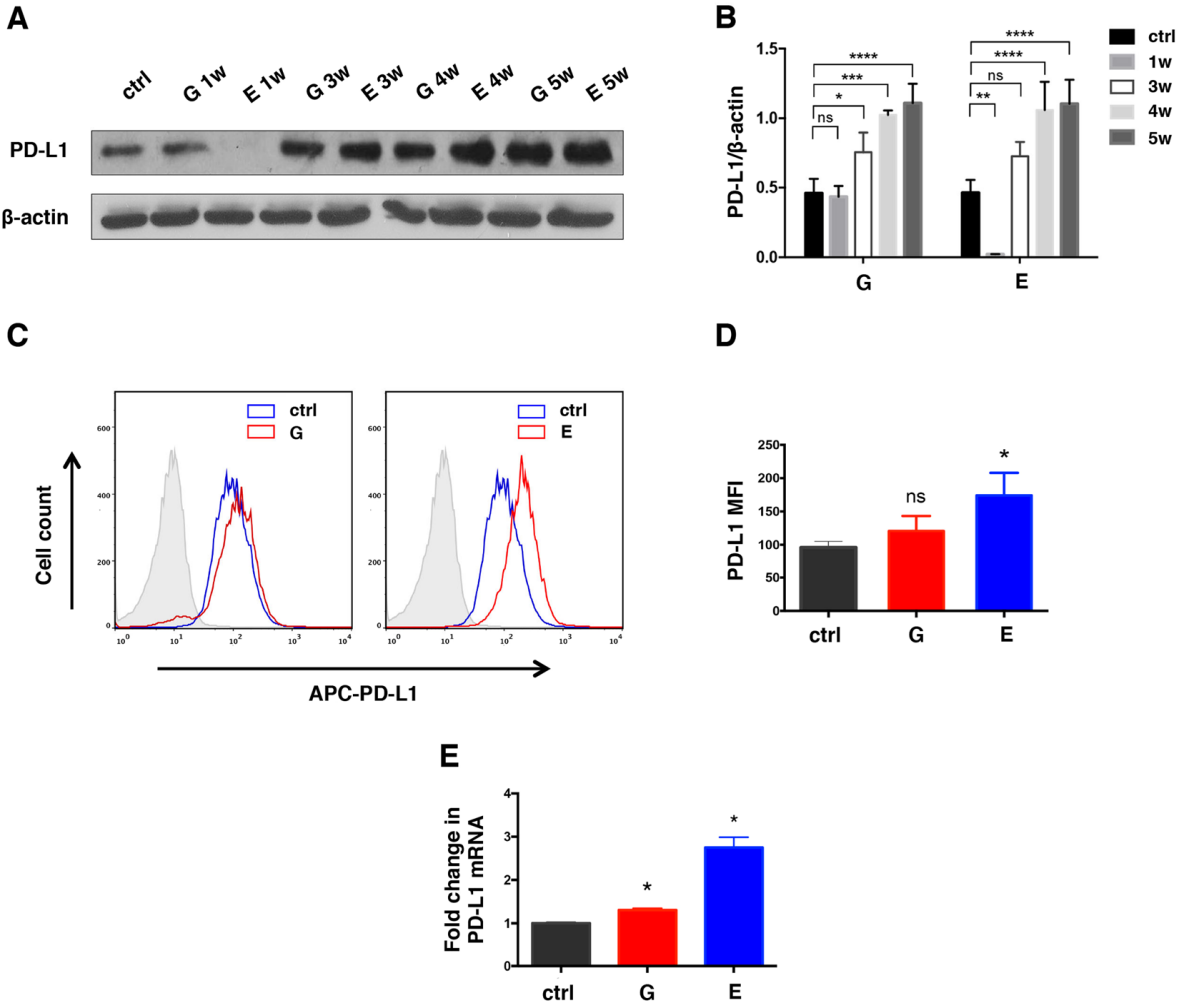
Constantly treatment with TKIs increases PD-L1 expression in H1975 (EGFR L858R/T790M). (**A,B**) Western blot and relative quantification of PD-L1 expression in H1975 treated with Gefitinib (5 μM) or Erlotinib (3 μM) for 1, 3, 4, 5 weeks. PD-L1 on the cell surface was also assessed by flow cytometry (**C,D**). (**E**) Quantitative PCR (qPCR) analysis of PD-L1 mRNA levels at 5 weeks. G, Gefitinib; E, Erlotinib; MFI, median fluorescence intensity; *p < 0.05; **p < 0.01; ***p < 0.001; ****p < 0.0001; ns, no significance.

To examine the influence of continuous exposure to TKIs on the PD-L1 expression in TKI-resistant NSCLC, H1975 cells which harbor the EGFR L858R/T790M double mutations were treated with Gefitinib or Erlotinib for 5 weeks. PD-L1 expression gradually upregulated with Gefitinib and Erlotinib treatment as showed by western blot (Fig. 1A,B). Though Erlotinib showed inhibitory effect on PD-L1 expression in the first week, PD-L1 quickly restored and increased at 3 weeks. Meanwhile, cell surface expression of PD-L1 also increased in Erlotinib treated cells as confirmed by the median florescence intensity (MFI) measured by flow cytometry while a slight upward trend was observed in Gefitinib treated cells (Fig. 1C,D). Consistently, we observed 1.3-fold and 2.8-fold increase in the levels of PD-L1 mRNA after treatment of Gefitinib and Erlotinib (Fig. 1E).

### Acquired resistance to TKIs promotes PD-L1 upregulation in HCC827

**Figure 2:**
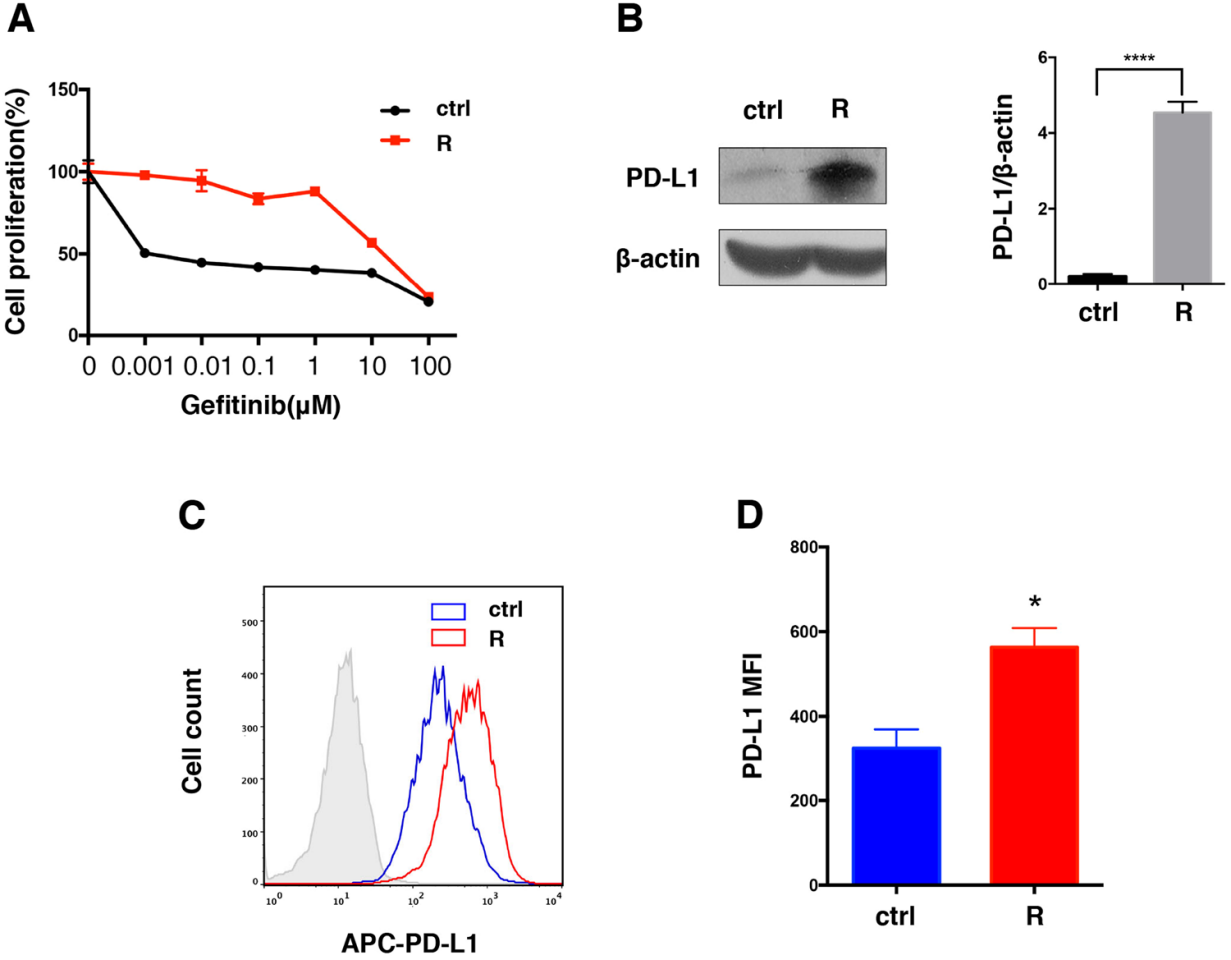
Long-term treatment with Gefitinib induces resistance and upregulates PD-L1 in HCC827 (EGFR Del 19). HCC827 became resistant to Gefitinib after continuous treatment for 6 months. (**A**) Dose-response curves comparing the parental and resistant cells treated with 0.001, 0.01, 0.1, 1, 10 and 100 μM Gefitinib for 72 hours. Cell viability was determined by MTT assay, Values were means ± SD of results. PD-L1 expression in resistant cells was assessed by Western blot (**B**) and flow cytometry (**C**,**D**). R, Gefitinib resistant. *p < 0.05; ****p < 0.0001.

The adaptive resistance to BRAF inhibitors was reported to result in the promotion of PD-L1 expression in melanoma cells^19^. To investigate the effect of acquired resistance on PD-L1 expression in NSCLC, HCC827 (EGFR Del 19), which is sensitive to TKIs therapy, was exposed to Gefitinib (2 μM) for more than 6 months. Finally, HCC827 became resistant to Gefitinib with increased IC50 values of 15.22 μM (Fig. 2A). Following the establishment of resistance, the level of PD-L1 expression increased significantly as showed by western blot (Fig. 2B). Flow cytometry also showed a 2.4-fold increase of PD-L1 on cell surface compared to parental cells (Fig. 2C,D).

### T cell function is impaired after the increase of PD-L1

**Figure 3:**
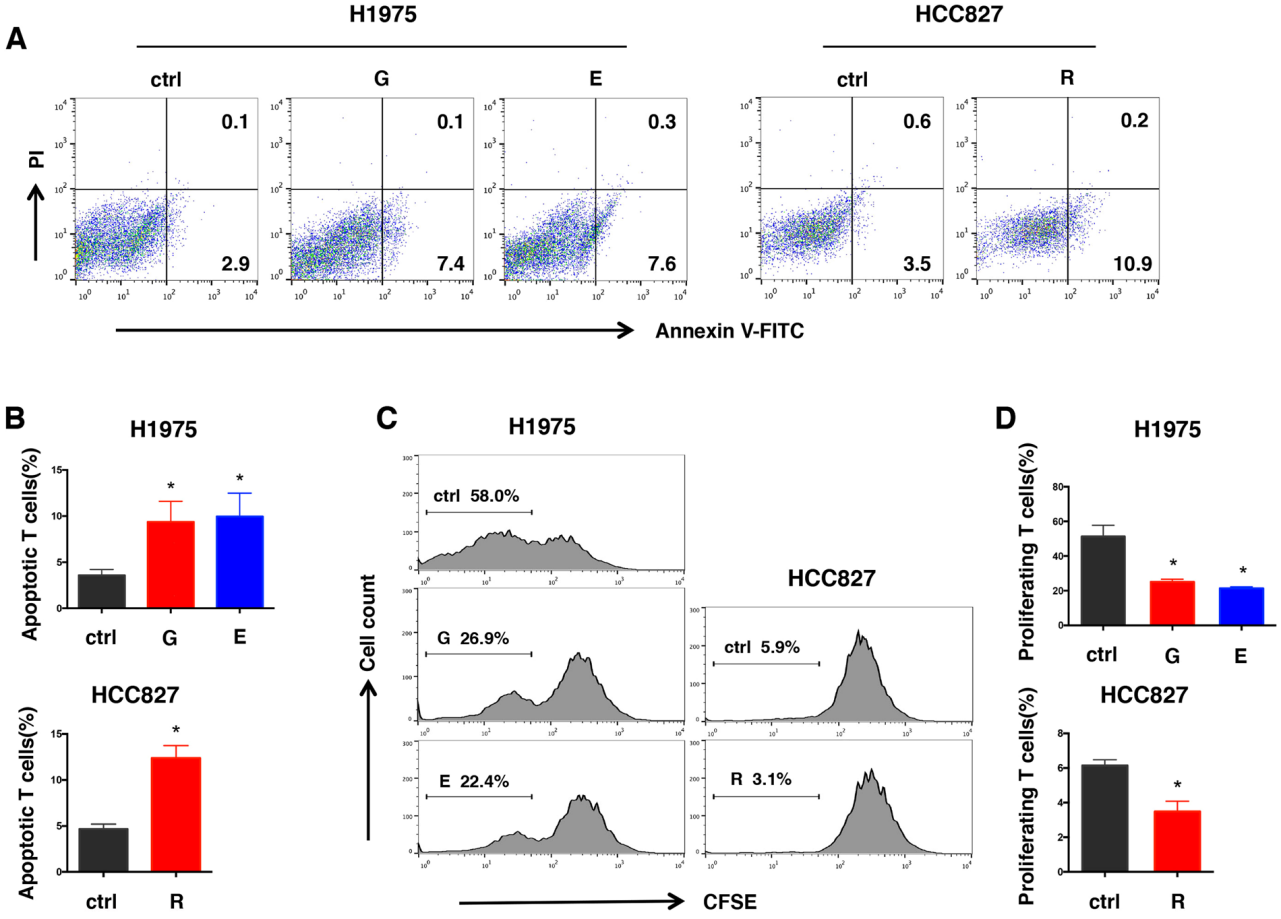
Increase of PD-L1 promotes apoptosis and inhibits proliferation of T cells. (**A**) Dot plot profile of T cells co-cultured with H1975 which were pretreated with Gefitinib or Erlotinib for 5 weeks and Gefitinib-resistant HCC827. Untreated tumor cells were set as control. After 24 hours coculture, T cells were harvested and stained with Annexin V and propidium iodide (PI) for apoptosis assay. (**B**) The flow cytometry results were presented as the percentage of apoptotic T cells. (**C**) Fluorescense histogram of carboxyfluorescein diacetate succinimidyl ester (CFSE) labeled T cells after co-culture with tumor cells mentioned above for 48 hours, untreated tumor cells were set as control. The gate population represents T cells that divided more than once. (**D**) The flow cytometry results were presented as the percentage of proliferating T cells. G, Gefitinib pretreated; E, Erlotinib pretreated; R, Gefitinib resistant; *p < 0.05.

To investigate the impact on T cells after the increase of PD-L1 on H1975 and HCC827, human peripheral blood mononuclear cells (PBMCs) were isolated from healthy donor and expanded *in vitro*. We evaluated both apoptosis and proliferation of T cells. Double labeling with Annexin V and PI staining showed that the percentage of apoptotic T cells slightly increased after co-culture with H1975 which has been treated with Gefitinib or Erlotinib for 5 weeks. Similarly, a slight increase of apoptotic T cells was observed in resistant HCC827 (Fig. 3A,B). When T cells were further characterized with proliferation-induced carboxyfluorescein diacetate succinimidyl ester (CFSE) dilution, we found that increased PD-L1 expression on H1975 significantly suppressed T cell proliferation. 58% of T cells proliferated after the co-culture with parental H1975, while only 26.9% and 22.4% in Gefitinib or Erlotinib treated cells. But very few T cells proliferated in both parental and resistant HCC827 (5.9% vs 3.1%) (Fig. 3C,D). Given that the PD-L1 expression in HCC827 was higher than that in H1975 (Supplemental Fig.S1), the parental HCC827 were able to markedly inhibit T cells proliferation, which suggested that certain PD-L1 protein level was sufficient to inhibit the proliferation of T cells.

### Upregulation of PD-L1 expression is not mediated by STAT3

**Figure 4:**
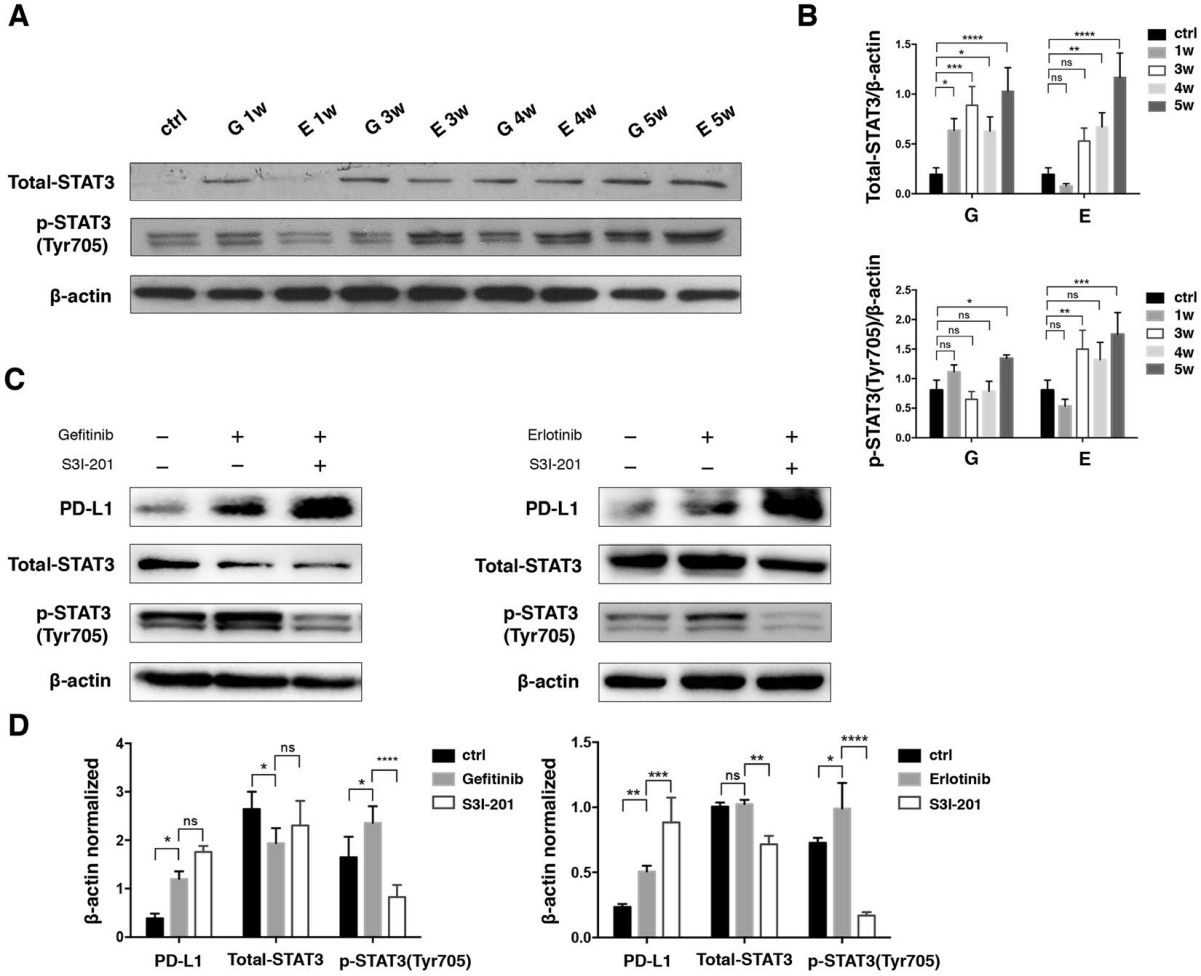
Inhibition of STAT3 activation increases PD-L1 dramatically. (**A,B**) Western blot analysis and relative quantification of signal transducer and activator of transcription 3 (STAT3) and its phosphorylation in H1975 treated with Gefitinib or Erlotinib for 1, 3, 4, 5 weeks. (**C,D**) Western blot analysis and relative quantification of PD-L1 expression in H1975 after inhibition of STAT3 with S3I-201 (40 μM) for 24 h at 5 weeks. *p < 0.05; **p < 0.01; ***p < 0.001; ****p < 0.0001; ns, no significance.

Activation of transcription factor STAT3 was reported to function in PD-L1 modulation and had a correlation to TKI resistance in NSCLC^19,20^. To elucidate the mechanism involved in PD-L1 upregulation, we assessed the STAT3 signaling pathway. The level of total STAT3 and p-STAT3 (Tyr705) exhibited increase trend during the TKIs treatment (Fig. 4A,B). S3I-201, a STAT3 inhibitor, was then used for further verification at 5 weeks. Interestingly, the PD-L1 dramatically increased after S3I-201 treatment regardless of the reduction of p-STAT3 (Tyr705) (Fig. 4C,D). Therefore, STAT3 was not involved in the induction of PD-L1 directly.

### The ERK1/2 pathway activation involves in the upregulation of PD-L1

**Figure 5:**
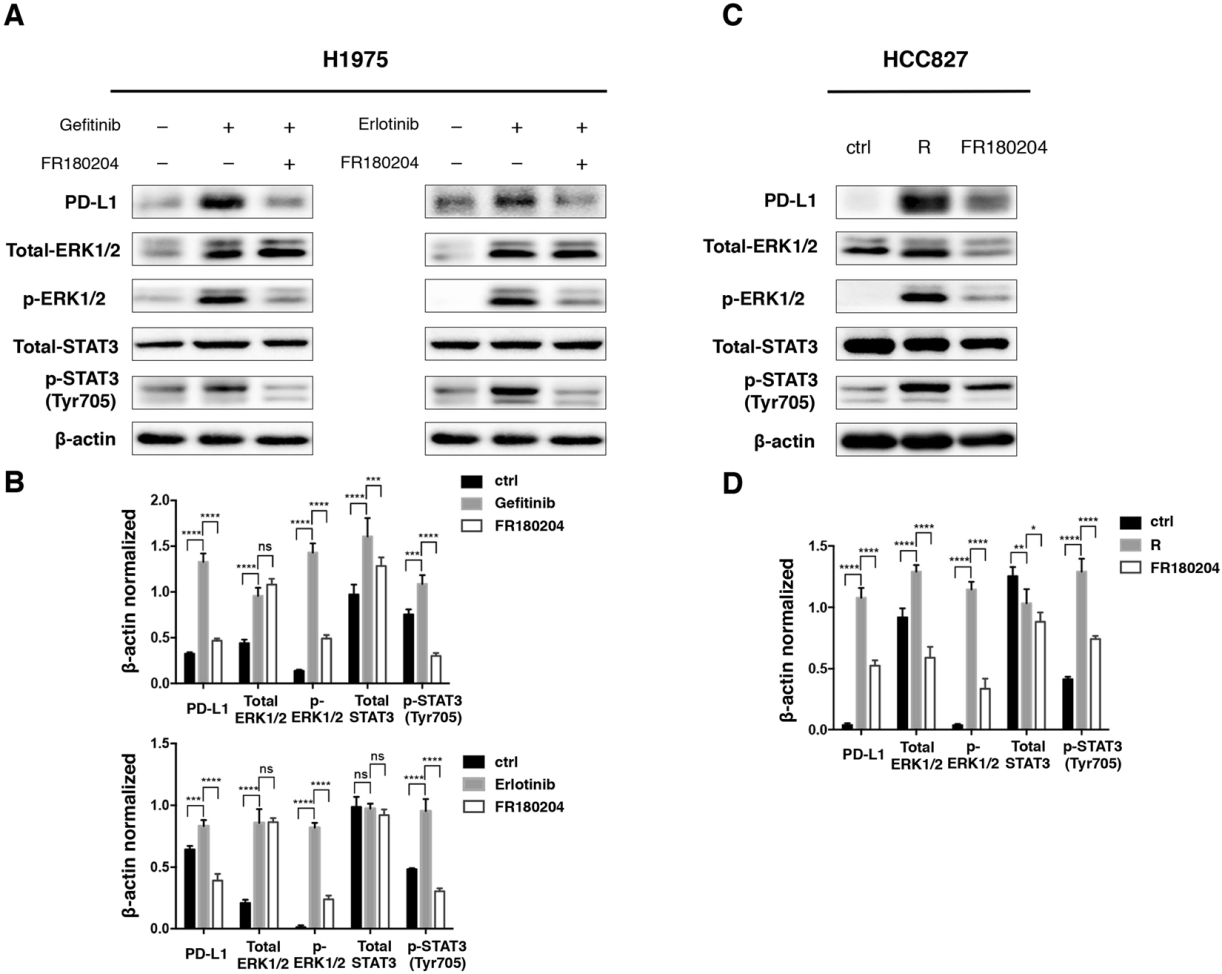
The activation of ERK1/2 pathway promotes PD-L1 expression. (**A,B**) Western blot analysis and relative quantification of p-ERK1/2 in H1975 treated with Gefitinib or Erlotinib for 5 weeks and PD-L1 expression after inhibition of ERK1/2 with FR 180204 (40 μM) for 24 h *in vitro*. STAT3 and its phosphorylated form were also analyzed. (**C,D**) Similarly, p-ERK1/2 and PD-L1 expression in resistant HCC827 were assessed by western blot after the treatment of FR 180204 for 24 h. R, Gefitinib resistant; *p < 0.05; **p < 0.01; ***p < 0.001; ****p < 0.0001; ns, no significance.

As extracellular-signal-regulated kinase (ERK1/2) was another critical pathway for PD-L1 regulation^19,21^, we decided to examine the possible contribution of the ERK1/2 pathway. Activation of ERK1/2 pathway was observed both in H1975 and HCC827 after TKIs treatment. Additionally, inhibiting ERK1/2 pathway with FR 180204 resulted in the decrease of PD-L1 in H1975, even though STAT3 phosphorylation was inhibited at the same time (Fig. 5A,B). Likewise, PD-L1 expression decreased apparently in resistant HCC827 after FR 180204 treatment (Fig. 5C,D). The result suggested ERK1/2 pathway played a key role in modulating PD-L1 expression in resistant lung cancer cells.

## Discussion

In the past decade, EGFR-TKIs yield significant success in the treatment of lung cancer. Routine test for EGFR mutation status characterizes NSCLC patients into different subgroups, resulting in encouraging efficacy and survival benefit in selected ones. However, primary or secondary drug resistance usually inevitably emerge over time, limiting the duration of response^22^. Along with the development of new effective agents targeting specific resistance mechanisms, a correlation between immune suppression and TKIs resistance is gradually being understood, indicating that immunotherapy can be a promising way to deal with the situation. Here, our study showed that PD-L1 expression was upregulated by continuous TKI treatment in resistant NSCLC resulting in persistent immune suppression.

An increasing body of evidence indicates that the activation of common oncogenic drivers in NSCLC (such as EGFR, ALK, and KRAS) is correlated with the induction of PD-L1 and corresponding inhibitors will reduce PD-L1 expression in initial treatment^16,23–25^. However, Jiang, X.*et al*. demonstrated that resistance to selective BRAF inhibition increased PD-L1 expression in melanoma^19^, which means there is a dynamic change of PD-L1 expression during the TKIs treatment. In our study, enduring use of EGFR TKIs in NSCLC induced not only drug resistance but also the increase of PD-L1. Notably a new correlation was revealed between the resistance of EGFR TKIs and PD-L1 expression in the long-term treatment setting.

Early studies reported that PD-L1 on tumor cells was sufficient for tumor immune evasion through directly inhibiting CD8+ T cell responses. Therapeutically blocking the PD-1 pathway leaded to immune reinvigoration and tumor regression^26–28^. So far, immune checkpoint inhibitor therapy has been considered a revolutionary treatment in lung cancer. After the success of CTLA-4 inhibitors in metastatic melanoma^29,30^, PD-L1 inhibitors show unprecedented responses in numerous clinical trials. PD-L1 expresses on a wide range of hematopoietic and non-hematopoietic cells including human cancer cells. High expression of PD-L1 is now regarded as a poor prognostic factor, contributing to immune escape and disease progression^31^. We found that PD-L1 upregulation mainly inhibited proliferation rather than promoted apoptosis of T cell, which would greatly restrain the antitumor immunity. The result suggested upregulation of PD-L1 is able to induce the immune suppress, indicating optional immune targeted therapy.

A number of preclinical studies have explored the possible mechanisms of PD-L1 modulation. The reported molecular mechanisms include MAPK pathway, PI3K-mTOR-S6 pathway, PI3K/PKB pathway, and JAK/STAT pathway, most of which have been extensively investigated in melanoma^19,32,33^. Our findings indicated that the increased phosphorylation of ERK1/2 pathway was engaged in inducing PD-L1 in resistant lung cancer. ERK1/2 pathway participates in cell proliferation, differentiation, and development and is critical for tumor survival. Meanwhile, previous study showed that activation of ERK1/2 signaling implicated in the resistance to EGFR kinase inhibitors^34^, suggesting an overlap in signaling mechanisms between TKI resistance and PD-L1 modulation. Moreover, clinical trials combining BRAF and MEK inhibitors with immune checkpoint blockades have been shown manageable safety profile and enhanced antitumor activity in melanoma^35,36^. As patients benefit temporarily from all the TKIs because of the acquired resistance, therapeutic synergy between EGFR-TKIs and PD-L1/PD-1 blockades or targeting downstream ERK1/2 signaling may prolong responses to these agents and delay the salvage treatment^37,38^.

STAT3 belongs to the STAT protein family, which involves various biological processes^39^. It was reported previously that STAT3 could bind to the PD-L1 promoter and modulated PD-L1 expression at the transcriptional level, and its inhibitors could decrease PD-L1 level in both parental and resistant melanoma^19^. Interestingly, the STAT3 inhibitor had no suppression effect on PD-L1 expression according to our study, conversely, strongly induced it, while ERK1/2 inhibitor suppressed STAT3 signaling at the same time but decreased PD-L1 expression successfully. Given that STAT3 siRNAs decreased PD-L1 expression by only 10–32% in two KRAS-mutant lung adenocarcinoma cell lines^23^, it is possible that STAT3 only partially involved. Considering the complexity of transcription and signaling networks, further investigation is needed to confirm the function of STAT3 in the regulation of PD-L1 in resistant lung cancer.

In conclusion, continuous TKIs treatment upregulates PD-L1 expression in resistant NSCLC, contributing to the suppression of T cell function and immune escape. ERK1/2 pathway plays a vital role in the regulation of PD-L1. Thus, reassessment of genetic alterations and signaling pathways after clinical progression is necessary for subsequent therapy. Based on our preclinical study, ERK pathway inhibitors, PD-L1/PD-1 inhibitors or combination strategies should be considered to overcome the TKI resistance and improve outcomes in NSCLC patients.

### Experimental procedures

#### Cell culture and Reagents

Human NSCLC cell lines including H1975 (EGFR L858R/T790M) and HCC827 (EGFR Del 19) were obtained from State Key Laboratory of Biotherapy, Sichuan University, and maintained with RPMI 1640 medium supplemented with 10% fetal bovine serum and 1% penicillin/streptomycin (SIGMA, USA) under a moist atmosphere of 5% CO_2_ at 37 °C. Gefitinib was bought from AstraZeneca (London, UK); Erlotinib was kindly provided by Roche Pharmaceuticals (Basel, Switzerland), while inhibitors including S3I-201 and FR180204 were bought from Selleck Chemicals. These drugs were dissolved in dimethyl sulfoxide (DMSO) and diluted in culture medium before use. Besides, primary rabbit antibodies against PD-L1 (CST#13684), ERK1/2 (CST#4695) and p-ERK1/2 (CST#4376) were from Cell Signaling Technology; rabbit antibodies to STAT3 (ab68153), p-STAT3 (Tyr705) (ab76315) were from Abcam while mouse antibody to β-actin was from Santa Cruz biotechnology.

#### Longtime exposure to TKI *in vitro*

H1975 was exposed (1 × 10^5^ cells/ml) continuously to 5 μM Gefitinib or 3 μM Erlotinib for 5 weeks. Total proteins were collected every week. 5 weeks later, the expression of PD-L1 was tested by flow cytometry while the total RNA was extracted as well. Meanwhile, HCC827 was constantly incubated (1 × 10^5^ cells/ml) with 2 μM Gefitinib for 6 months.

#### Western blot analyses

Tumor cells were harvested in cold RIPA lysis buffer (Beyotime, China) containing protease inhibitor cocktail and 1 mM phenylmethylsulfonyl fluoride. And the protein concentration was detected by bicinchoninic acid (BCA) protein assay. After cell lysates were separated in the SDS-PAGE Gels, they were transferred to the polyvinylidene difluoride (PVDF) membrane (Millipore, USA), which was then blocked with 5% non-fat milk. After the membrane was cut into small pieces, they were incubated in different primary antibodies at a 1:1000 dilution overnight at 4 °C. Then the membranes reacted with the anti-rabbit or mouse IgG conjugated with horseradish peroxidase (HRP) (Beyotime, China). Finally, visible bands were obtained through the enhanced chemiluminescence (ECL) detection.

#### RNA extraction and analysis by quantitative real-time PCR

Total RNA was extracted and purified using RNA simple Total RNA Kit (TIANGEN BIOTECH, China) according to the manufacturer’s instructions. The RNA samples were reverse-transcribed into cDNA with the PrimeScript^TM^ RT reagent Kit (TAKARA, Japan). Quantitative real-time PCR was conducted with Bio-rad CFX manager and SsoAdvanced SYBR Green Supermix (Bio-rad, USA) as the detection reagent. All samples were analyzed in triplicate for each primer set. The primer sequences of PCR were as follows: hPD-L1, sense 5-GGAATTGTCTCAGAATGGTC-3 and antisense 5-GTAGTTGCTTCTAGGAAGGAG-3; hGAPDH, sense 5-GAAGGTGAAGGTCGGAGT-3 and antisense 5-GAAGATGGTGATGGGATTTC-3. RT-PCR amplification was performed 40 cycles with DNA denaturation at 95 °C for 5 s and annealing/extension at 60 °C for 20 s. Target gene expression was quantified with the relative quantification method.

#### Flow cytometry

Cells were collected and dissociated as single cells, then washed with 0.1% bovine serum albumin in PBS (PBSA) and stained with APC-conjugated anti-human CD274 antibody (Clone 29E.2A3, Biolegend) or isotype-matched control antibody (clone MOPC-21, Biolegend). After 30 minutes of incubation, cells were washed and resuspended in PBS, then performed on a BD Biosciences FACSCalibur cytometer. Data were analyzed using FlowJo software program.

### T cell apoptosis and proliferation assay

#### T cell preparation and co-culture system

Human peripheral blood mononuclear cells (PBMCs) were isolated from the blood of healthy volunteer donors by Ficoll Paque density centrifugation. T cells were selected by magnet (DynaMag™-50 Magnet, Life Technologies) and stimulated with recombinant human IL-2 (PeproTech, 30 U/mL) and Dynabeads Human T-activator CD3/CD28 beads (Gibco, 4 × 10^7^ beads/mL) (bead-to-cell ratio 1:1). H1975, which were already treated with Gefitinib or Erlotinib for 5 weeks, and Gefitinib-resistant HCC827 cells were seeded in 24-well plates (5 × 10^4^ cells/well) with parental cells as control and cultured in plain medium (with serum) overnight. Then isolated T cells were added to each well at a ratio of 2:1 (T cells: tumor cells) and co-cultured for 24 hours. Parental tumor cells were set as control.

#### Apoptosis assay

Suspended cells from the co-culture system were collected and stained with anti-human APC-conjugated CD3 antibody (clone HIT3a, Biolegend) for 30 minutes on ice. Then washed T cells were counterstained with FITC Annexin V and PI for 15 min at room temperature (25 °C) according to the manufacturer’s instructions of FITC Annexin V Apoptosis Detection Kit I (BD Pharmingen). Finally the FITC Annexin V and PI signal of T cells were analyzed by flow cytometry.

#### Proliferation assay

Expanded T cells were labeled with carboxyfluorescein diacetate succinimidyl ester (CFSE, Biolegend) prior to co-culture with tumor cells (described above). After 48 hours, suspended cells were harvested and counterstained with anti-human CD3-APC antibody. The CFSE signal of T cells was analyzed by flow cytometry.

#### Cell viability assay

HCC827 were plated in 96-well at a concentration of 2 × 10^3^ cells per well. Cells were allowed to grow for 24 hours and then treated with Gefitinib at various concentrations (0.001, 0.01, 0.1,1, 10 and 100 μM), in six replicates. After 72 hours of treatment, 20 μL MTT (3-(4,5 dimethylthiazol-2-yl)-2,5 diphenyltetrazolium bromide) (Sigma-Aldrich) (5 mg/mL) was added to each well, incubated at 37 °C for 4 hours and terminated with 150 μL of dimethyl sulfoxide (Sigma-Aldrich). Cell viability was finally determined with an ELISA reader (Bio-Rad) at 570 nm. The 50% inhibitory concentration (IC50) values were calculated using a proper analysis (nonlinear regression-curve fit).

#### Statistical Analysis

Experiments were all repeated in triplicate. For significance analysis of PD-L1 expression (flow cytometry, real-time PCR), western blot analysis and proliferation and apoptosis of T cells, *t* test for two groups and one-way ANOVA Tukey test followed by Dunnett’s multiple comparison test for multiple groups were performed using GraphPad Prism 7. All data were summarized and presented as means ± SD. Statistical significance was defined as: “ns”*p* > 0.05, “*”*p* < 0.05, “**”*p* < 0.01, “***”*p* < 0.001 and “****”*p* < 0.0001.

#### Ethics Statement

All experiments involving human tissue samples were performed in accordance with relevant guidelines and regulations approved by medical ethics committee of Sichuan University. Informed consent was obtained from all healthy donors.

## Supplementary information

supplementary figure S1–6

## References

[1] Siegel RL, Miller KD, Jemal A (2018). Cancer statistics, 2018. CA: a cancer journal for clinicians.

[2] Miller KD (2016). Cancer treatment and survivorship statistics, 2016. CA: a cancer journal for clinicians.

[3] Zhou C (2011). Erlotinib versus chemotherapy as first-line treatment for patients with advanced EGFR mutation-positive non-small-cell lung cancer (OPTIMAL, CTONG-0802): a multicentre, open-label, randomised, phase 3 study. The Lancet Oncology.

[4] Fukuoka M (2011). Biomarker analyses and final overall survival results from a phase III, randomized, open-label, first-line study of gefitinib versus carboplatin/paclitaxel in clinically selected patients with advanced non-small-cell lung cancer in Asia (IPASS). Journal of clinical oncology: official journal of the American Society of Clinical Oncology.

[5] Solomon BJ (2014). First-line crizotinib versus chemotherapy in ALK-positive lung cancer. The New England journal of medicine.

[6] da Cunha Santos G, Shepherd FA, Tsao MS (2011). EGFR mutations and lung cancer. Annual review of pathology.

[7] Jimeno A, Hidalgo M (2006). Pharmacogenomics of epidermal growth factor receptor (EGFR) tyrosine kinase inhibitors. Biochimica et biophysica acta.

[8] Sequist LV, Bell DW, Lynch TJ, Haber DA (2007). Molecular predictors of response to epidermal growth factor receptor antagonists in non-small-cell lung cancer. Journal of clinical oncology: official journal of the American Society of Clinical Oncology.

[9] Sequist LV (2011). Genotypic and histological evolution of lung cancers acquiring resistance to EGFR inhibitors. Science translational medicine.

[10] Yun CH (2008). The T790M mutation in EGFR kinase causes drug resistance by increasing the affinity for ATP. Proceedings of the National Academy of Sciences of the United States of America.

[11] Sharma SV, Bell DW, Settleman J, Haber DA (2007). Epidermal growth factor receptor mutations in lung cancer. Nature reviews. Cancer.

[12] Gandara DR (2014). Acquired resistance to targeted therapies against oncogene-driven non-small-cell lung cancer: approach to subtyping progressive disease and clinical implications. Clin Lung Cancer.

[13] Soria J-C (2015). Gefitinib plus chemotherapy versus placebo plus chemotherapy in EGFR-mutation-positive non-small-cell lung cancer after progression on first-line gefitinib (IMPRESS): a phase 3 randomised trial. The Lancet Oncology.

[14] Nishie K (2012). Epidermal growth factor receptor tyrosine kinase inhibitors beyond progressive disease: a retrospective analysis for Japanese patients with activating EGFR mutations. J Thorac Oncol.

[15] Park, K. *et al*. First-Line Erlotinib Therapy Until and Beyond Response Evaluation Criteria in Solid Tumors Progression in Asian Patients With Epidermal Growth Factor Receptor Mutation-Positive Non-Small-Cell Lung Cancer: The ASPIRATION Study. *JAMA oncology*, 1–8 (2015). 10.1001/jamaoncol.2015.4921 26720423

[16] Akbay EA (2013). Activation of the PD-1 pathway contributes to immune escape in EGFR-driven lung tumors. Cancer discovery.

[17] Keir ME, Butte MJ, Freeman GJ, Sharpe AH (2008). PD-1 and its ligands in tolerance and immunity. Annual review of immunology.

[18] Tang J (2015). Continuous exposure of non-small cell lung cancer cells with wild-type EGFR to an inhibitor of EGFR tyrosine kinase induces chemoresistance by activating STAT3. Int J Oncol.

[19] Jiang X, Zhou J, Giobbie-Hurder A, Wargo J, Hodi FS (2013). The activation of MAPK in melanoma cells resistant to BRAF inhibition promotes PD-L1 expression that is reversible by MEK and PI3K inhibition. Clinical cancer research: an official journal of the American Association for Cancer Research.

[20] Lee HJ (2014). Drug resistance via feedback activation of Stat3 in oncogene-addicted cancer cells. Cancer cell.

[21] Atefi M (2014). Effects of MAPK and PI3K pathways on PD-L1 expression in melanoma. Clinical cancer research: an official journal of the American Association for Cancer Research.

[22] Daniele Focosi, E. Resistance to Tyrosine Kinase Inhibitors. *Springer Nature* **10** (2016).

[23] Sumimoto H, Takano A, Teramoto K, Daigo Y (2016). RAS-Mitogen-Activated Protein Kinase Signal Is Required for Enhanced PD-L1 Expression in Human Lung Cancers. PloS one.

[24] Li D, Zhu X, Wang H, Li N (2017). Association between PD-L1 expression and driven gene status in NSCLC: A meta-analysis. European journal of surgical oncology: the journal of the European Society of Surgical Oncology and the British Association of Surgical Oncology.

[25] Ota K (2015). Induction of PD-L1 Expression by the EML4–ALK Oncoprotein and Downstream Signaling Pathways in Non–Small Cell Lung Cancer. Clinical Cancer Research.

[26] Juneja VR (2017). PD-L1 on tumor cells is sufficient for immune evasion in immunogenic tumors and inhibits CD8 T cell cytotoxicity. The Journal of experimental medicine.

[27] Brahmer JR (2010). Phase I study of single-agent anti-programmed death-1 (MDX-1106) in refractory solid tumors: safety, clinical activity, pharmacodynamics, and immunologic correlates. Journal of clinical oncology: official journal of the American Society of Clinical Oncology.

[28] Brahmer JR (2012). Safety and activity of anti-PD-L1 antibody in patients with advanced cancer. The New England journal of medicine.

[29] Robert C (2013). Efficacy and safety of retreatment with ipilimumab in patients with pretreated advanced melanoma who progressed after initially achieving disease control. Clinical cancer research: an official journal of the American Association for Cancer Research.

[30] Prieto PA (2012). CTLA-4 blockade with ipilimumab: long-term follow-up of 177 patients with metastatic melanoma. Clinical cancer research: an official journal of the American Association for Cancer Research.

[31] Mu CY, Huang JA, Chen Y, Chen C, Zhang XG (2011). High expression of PD-L1 in lung cancer may contribute to poor prognosis and tumor cells immune escape through suppressing tumor infiltrating dendritic cells maturation. Medical oncology.

[32] Han JJ (2016). Change in PD-L1 Expression After Acquiring Resistance to Gefitinib in EGFR-Mutant Non-Small-Cell Lung Cancer. Clin Lung Cancer.

[33] Abdelhamed S, Ogura K, Yokoyama S, Saiki I, Hayakawa Y (2016). AKT-STAT3 Pathway as a Downstream Target of EGFR Signaling to Regulate PD-L1 Expression on NSCLC cells. J Cancer.

[34] Ercan D (2012). Reactivation of ERK signaling causes resistance to EGFR kinase inhibitors. Cancer discovery.

[35] Wargo JA, Cooper ZA, Flaherty KT (2014). Universes collide: combining immunotherapy with targeted therapy for cancer. Cancer discovery.

[36] Hu-Lieskovan, S. *et al*. Improved antitumor activity of immunotherapy with BRAF and MEK inhibitors in BRAFV600E melanoma. *Science translational medicine* **7** (2015). 10.1126/scitranslmed.aaa4691 PMC4765379 25787767

[37] Deken MA (2016). Targeting the MAPK and PI3K pathways in combination with PD1 blockade in melanoma. Oncoimmunology.

[38] Rotow J, Bivona TG (2017). Understanding and targeting resistance mechanisms in NSCLC. Nature reviews. Cancer.

[39] Miklossy G, Hilliard TS, Turkson J (2013). Therapeutic modulators of STAT signalling for human diseases. Nature reviews. Drug discovery.

