# Supplementary material for: Continuous targeted kinase inhibitors treatment induces upregulation of PD-L1 in resistant NSCLC
Source: Sci Rep. 2019 Mar 6;9:3705. doi: 10.1038/s41598-018-38068-3 (PMC6403384; doi:10.1038/s41598-018-38068-3)
Supplement: Supplementary file 1 — supplementary figure S1–6 [file 41598_2018_38068_MOESM1_ESM.docx]

Supplementary Information

**Continuous targeted kinase inhibitors treatment induces upregulation of PD-L1 in resistant NSCLC**

Li Jiang^1^, Fuchun Guo^1,2^, Xiaoke Liu^1^, Xiaoyu Li^1^, Qing Qin^1^, Pei Shu^1^, Yi Li^1^, and Yongsheng Wang^1,2,^*

^1^Departments of Thoracic Oncology, Cancer Center, West China Hospital, Chengdu, Sichuan Province, 610041 China

^2^State Key Laboratory of Biotherapy, Sichuan University, Chengdu, Sichuan Province, 610041 China

*To whom correspondence should addressed: [wangys@scu.edu.cn](mailto:wangys@scu.edu.cn)

**Supplementary Fig. S1** PD-L1 expression in NSCLC with mutated or wild-type EGFR

Flow cytometry analysis of PD-L1 expression in NSCLC harboring mutated EGFR (H1975, HCC827) or wild-type EGFR (H1299, H292).


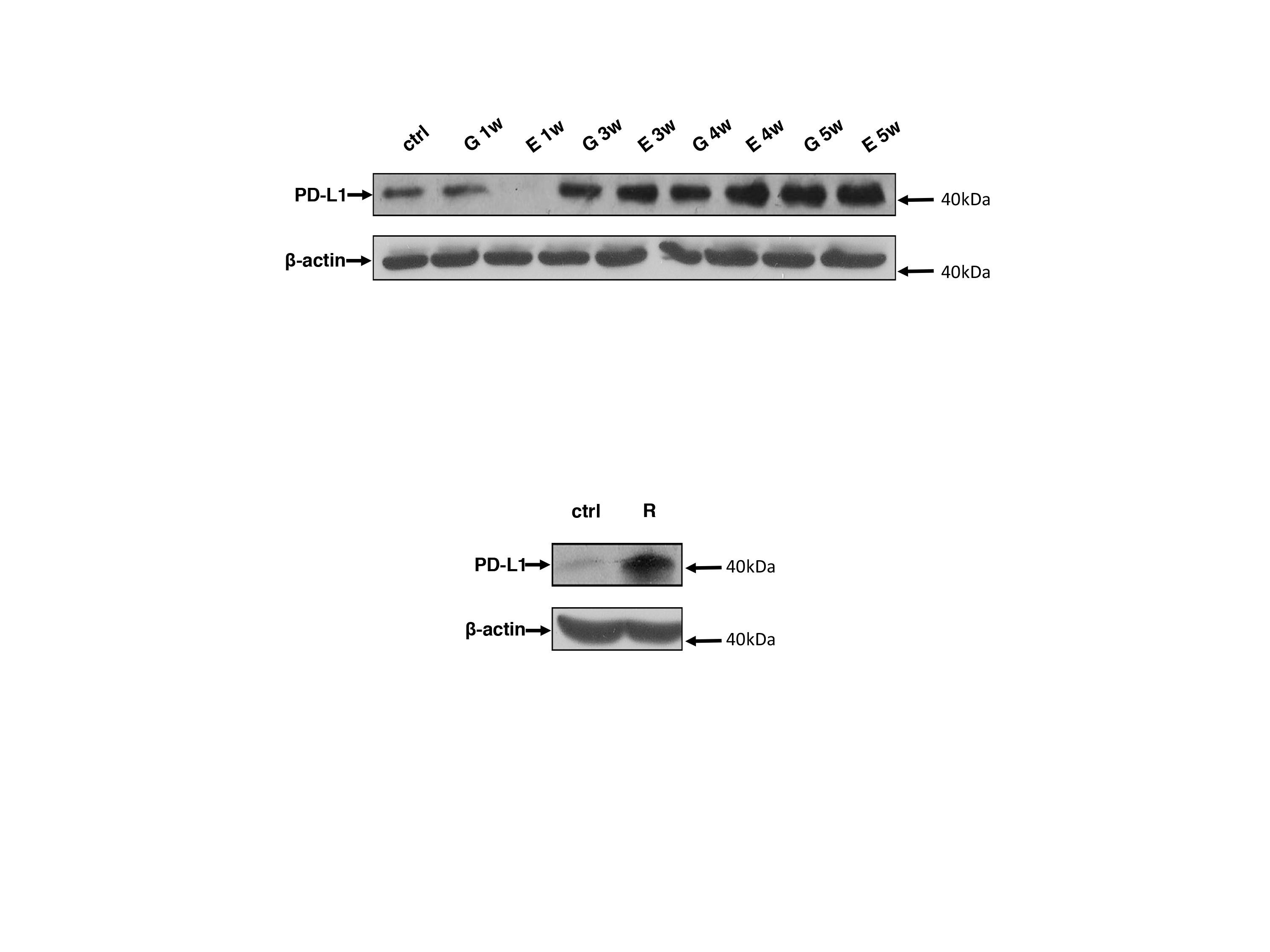


**Supplementary Fig. S2** Constantly treatment with Gefitinib or Erlotinib increases PD-L1 expression in H1975

PD-L1 expression in H1975 after treatment with Gefitinib (5 μM) or Erlotinib (3 μM) for 1, 3, 4, 5 weeks. G, Gefitinib; E, Erlotinib. These cropped blots are used in the main figure (Figure 1) and these full-length blots are included in the supplementary figure.


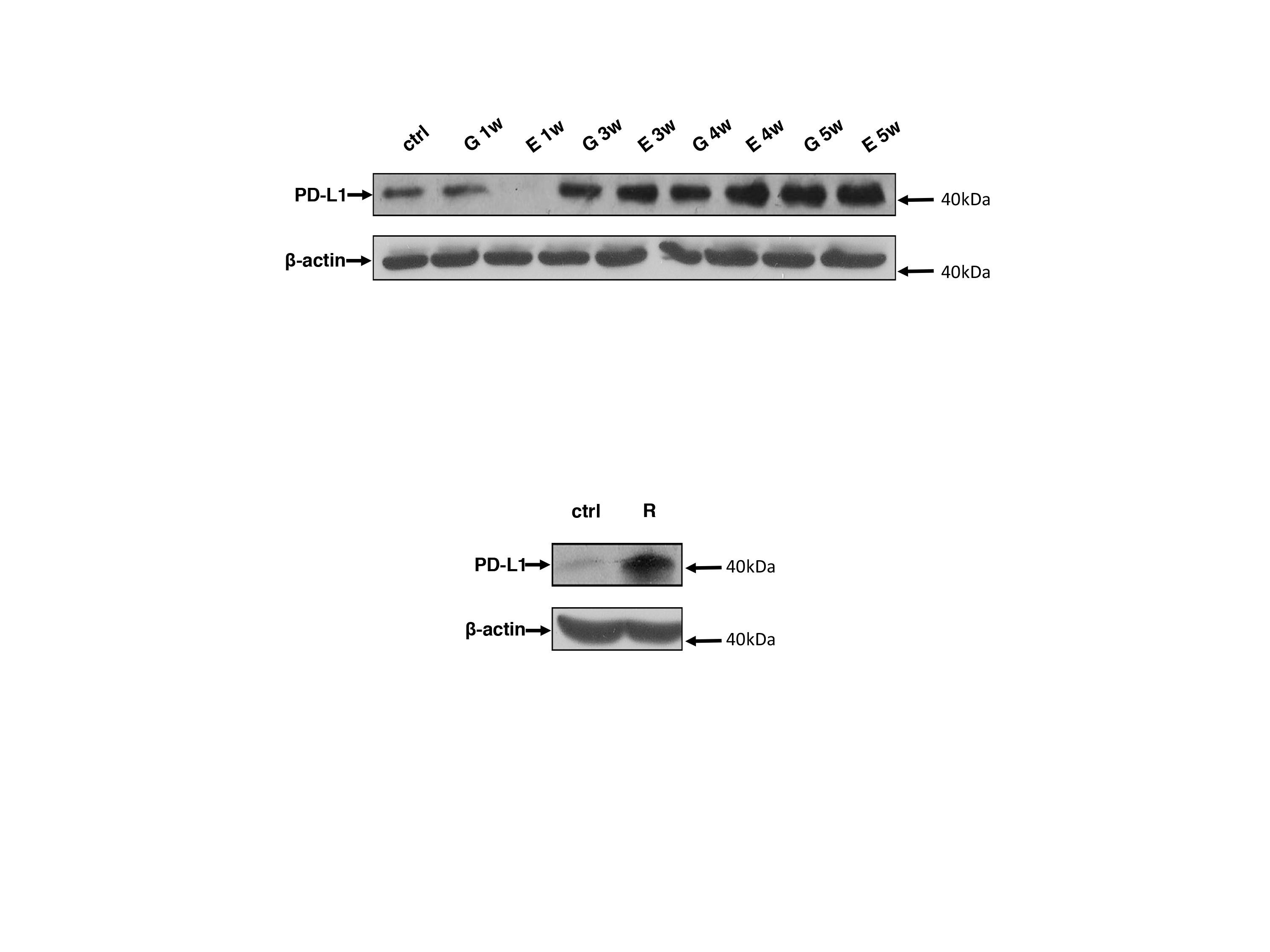


**Supplementary Fig. S3** Acquired resistance induces PD-L1 upregulation in HCC827

Expression analysis of PD-L1 in parental and resistant HCC827. R, Gefitinib resistant. These cropped blots are used in the main figure (Figure 2) and these full-length blots are included in the supplementary figure.


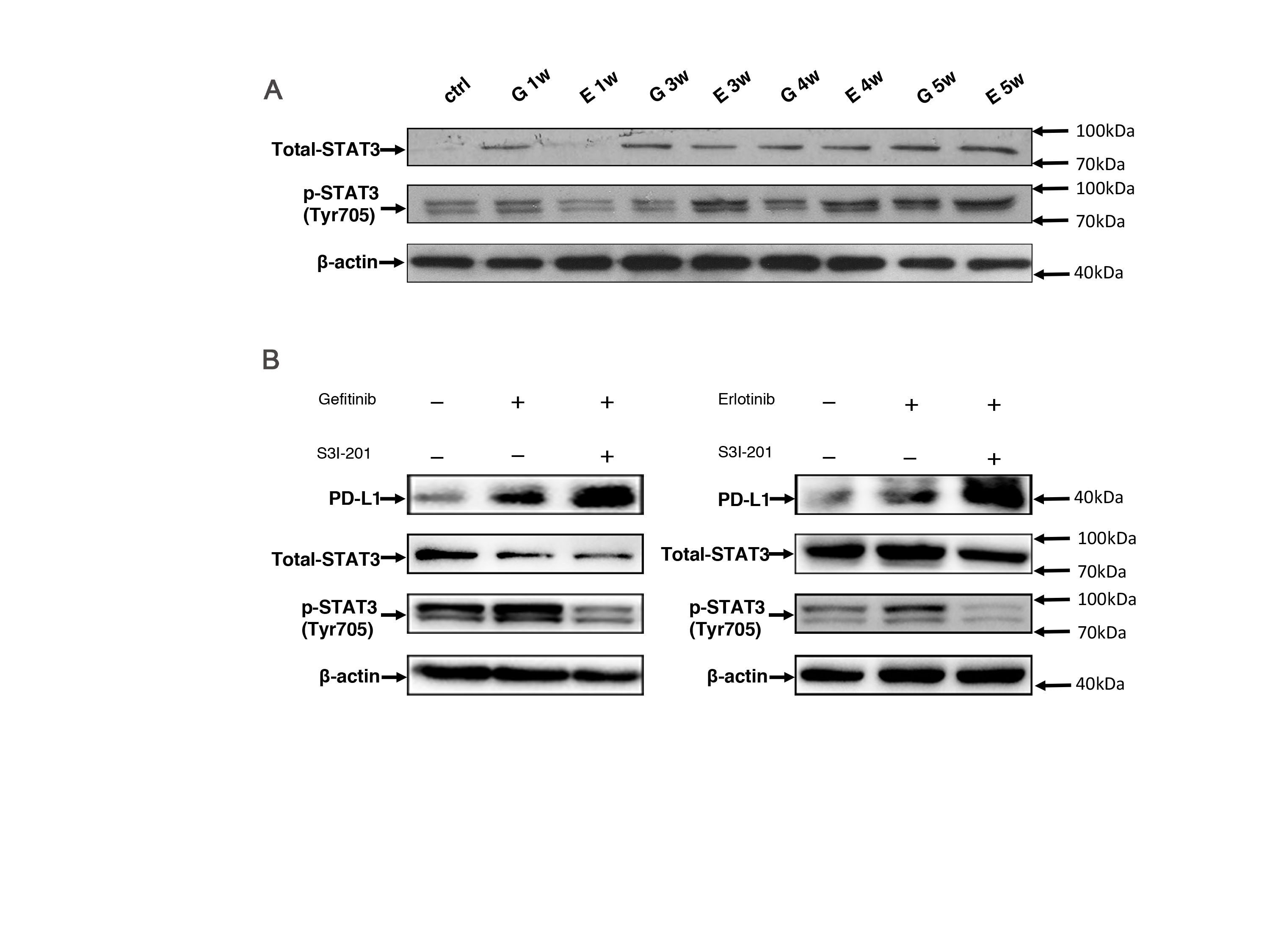


**Supplementary Fig. S4** Inhibition of STAT3 activation increases PD-L1 dramatically

(A)Western blot analysis of signal transducer and activator of transcription 3 (STAT3) and its phosphorylation in H1975 treated with Gefitinib or Erlotinib for 1, 3, 4, 5 weeks. (B) Western blot analysis of PD-L1 expression in H1975 after inhibition of STAT3 with S3I-201 (40μM) at 5 weeks. G, Gefitinib; E, Erlotinib. These cropped blots are used in the main figure (Figure 4) and these full-length blots are included in the supplementary figure.


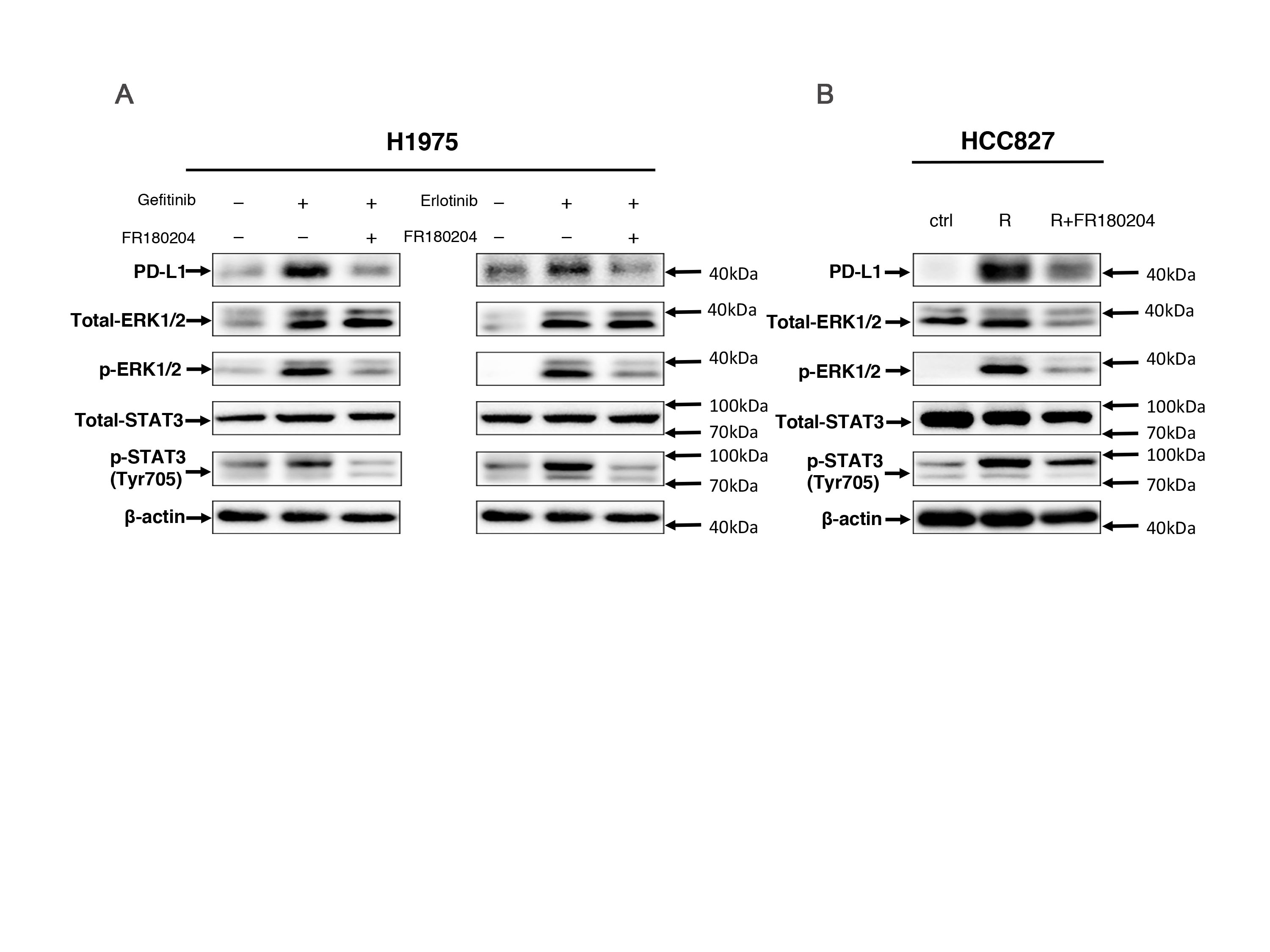


**Supplementary Fig. S5** The activation of ERK1/2 pathway promotes PD-L1 expression.

(A)Western blot analysis of p-ERK1/2 in H1975 treated with Gefitinib or Erlotinib for 5 weeks and PD-L1 expression after inhibition of ERK1/2 with FR 180204 (40 μM) in vitro. STAT3 and its phosphorylated form were also analyzed. (B) Similarly, p-ERK1/2 and PD-L1 expression in resistant HCC827 were assessed by western blot after the treatment of FR 180204. R, Gefitinib resistant. These cropped blots are used in the main figure (Figure 5) and these full-length blots are included in the supplementary figure.


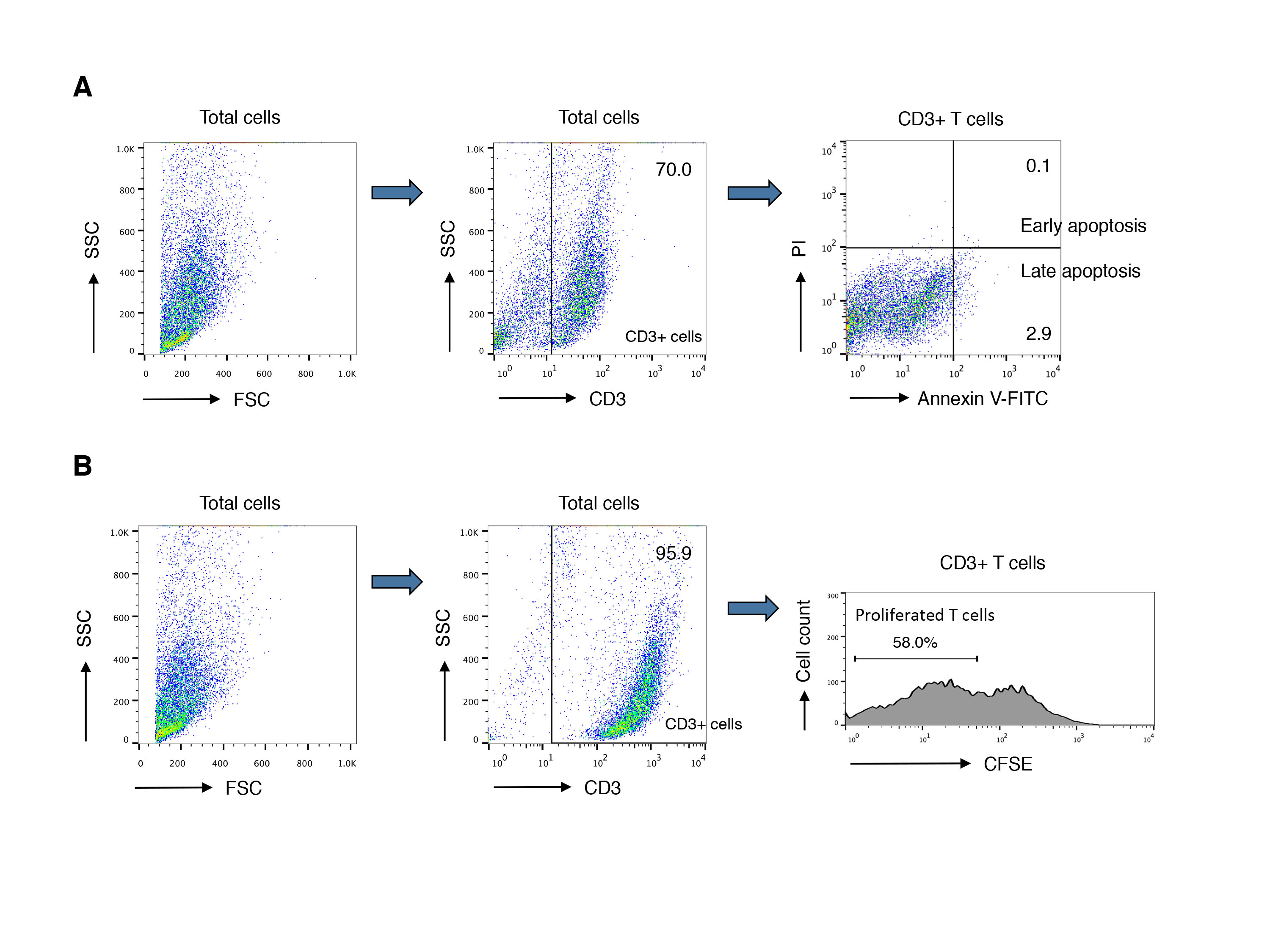


**Supplementary Fig. S6** Gating strategy for flow cytometry analysis.

(A) In this sample gating of apoptosis analysis, cells were first gated by CD3 and then the CD3 positive gate was further analyzed for their fluoresce of FITC Annexin V and PI. FITC Annexin V negative and PI negative indicate viable cells; FITC Annexin V positive and PI negative indicate cells in early apoptosis; FITC Annexin V positive and PI positive indicate cells in late apoptosis. The sum of cells in early apoptosis and late apoptosis represents the apoptotic T cells. (B) In this sample gating of proliferation analysis, cells were first gated by CD3 and then the CD3 positive gate was further analyzed for their fluoresce of CFSE. The signal of CFSE was diluted when T cells divided more than once. The gate population represents proliferated T cells.
